# Complete mitochondrial genome of Simao pine caterpillar moth, *Dendrolimus kikuchii* (Lepidoptera: Lasiocampidae) and the related phylogenetic analysis

**DOI:** 10.1080/23802359.2017.1372725

**Published:** 2017-09-05

**Authors:** Jian-Hong Liu, Jun-Qing Fu, Li-Ying Yang, Ping-Fan Jia

**Affiliations:** a Key Laboratory of Forest Disaster Warning and Control in Yunnan Province, Southwest Forestry University, Kunming, China;; b School of Nursing and Rehabilitation, Xinyu University, Xinyu, China

**Keywords:** *Dendrolimus kikuchii*, mitochondrial genome, molecular phylogeny

## Abstract

*Dendrolimus kikuchii*, the Simao pine caterpillar moth is an economically important forest pest causing great damage to coniferous trees in south China. The whole mitogenome for the moth was sequenced using next-generation sequencing technology. The moth has a total length of 15,422 bp in mitogenome size. The nucleotide composition is biased toward adenine and thymine, accounting for 79.2%: A = 40.7%, T = 38.5%, G = 8.1%, and C = 12.7%. The phylogeny showed that *D. kikuchii* was closely grouped with *D. punctatus, D. tabulaeformis, Euthrix laeta*, and *Trabala vishnou guttata* to form the monophyletic clad for the Lasiocampidae family of Bombycoidea superfamily with strong nodal support.

*Dendrolimus kikuchii,* the Simao pine caterpillar moth, belongs to Lasiocampidae family in the superfamily of Bombycoidea. This species is widespread across the Provinces of south China, including Yunnan, Guizhou, Sichuan, Guangdong, Guangxi, Taiwan, Hunan, Hubei, Jiangxi, Fujian, Zhejiang, Anhui (Tong and He 2009). Its larvae feed on such coniferous trees as *Pinus kesiya var. langbianensis*, *P. yunnanensis*, *P. armandii*, *P. massoniana*, *P. taiwanensis*, *P. fenzeliana*, *Cedrus deodara*, and *Keteleeria evelyniana* and cause significant economic loss during larval outbreaks (Kong et al. 2001, 2011; Dai et al. 2012).

The whole mitochondrial genome of *D. kikuchii* was identified based on next-generation sequencing technology in the present study. Adult samples for the species were collected from Yongping town (23.42°N, 100.40°E), located in Jinggu County in Yunnan Province of southwest China. Specimen was deposited in Key Laboratory of Forest Disaster Warning and Control in Yunnan Province, Kunming, China. Mitochondrial genomic DNA was extracted according to the manufacturer’s instruction in the DNeasy Blood and Tissue kit (Qiagen, Hilden, Germany) for animal tissue. Genome shotgun reads were sequenced with the Illumina HiSeq platform and complete mitochondrial genome was assembled with high coverage using Illumina sequencing data. The sequence was preliminarily aligned using the CLUSTAL X program (Thompson et al. 1997). The complete mitogenome of 16 species in Bombycoidea superfamily was clustered in MEGA 7 (Kumar et al. 2016). The phylogenetic relationship was inferred by using the maximum likelihood method based on the Tamura-Nei model (Tamura and Nei 1993). The tree inferred from 500 replicates was taken to represent the phylogeny of the species analysed (Felsenstein 1985).

The whole mitogenome of *D. kikuchii* sequence is a closed circular double-strand DNA molecule (GenBank accession number: MF596492). The moth has a total length of 15,422 bp in mitogenome size which is well within the range in the completely sequenced mitogenomes for 16 species in the superfamily of Bombycoidea available with the size from 15,236 bp in *Actias selene* to 15,566 bp in *Antheraea pernyi*. The gene content is typical of other insect mitogenomes within Bombycoidea superfamily, consisting of 22 tRNA genes, 13 PCGs, two rRNA genes, and a major non-coding region known as the CR. The A + T content is a parameter which was usually used in the investigation of the nucleotide-compositional behaviour of mitogenome (Hassanin et al. 2005; Wei et al. 2010; Song et al. 2016). The nucleotide composition of the whole mtDNA sequence for *D. kikuchii* is biased toward A and T (accounting for 79.2%: A = 40.7%, T = 38.5%, G = 8.1%, and C = 12.7%). This bias is well within the range observed in the completely sequenced mitogenome of 16 species in Bombycoidea superfamily, from 78.6% in *Actias artemis aliena* to 81.8% in *Manduca sexta*. Mediterranean fruit fly, *Ceratitis capitate* (Diptera: Tephritidae), was taken as an outgroup and the phylogenetic position of *D. kikuchii* was closely clustered with *D. punctatus, D. tabulaeformis, Euthrix laeta, Trabala vishnou guttata* and formed the monophyletic clad for Lasiocampidae family of the superfamily of Bombycoidea with strong nodal support (Figure 1). Within Bombycoidea superfamily, the clad for the Sphingidae family, consisting of *Manduca sexta, Sphinx morio, Notonagemia analis scribae* and *Ampelophaga rubiginosa*, was recovered as the sister group of the Saturniidae family clade, including *Antheraea pernyi, Antheraea yamamai, Antheraea assama, Actias selene, Actias artemis alien, Eriogyna pyretorum, Attacus atlas* which were highly supported (Figure 1). In conclusion, the whole mitogenome of *D. kikuchii* was identified for the first time in this study and can provide essential DNA molecular data for further phylogenetic and evolutionary analysis for Bombycoidea superfamily of Lepidoptera order.

**Figure 1.**
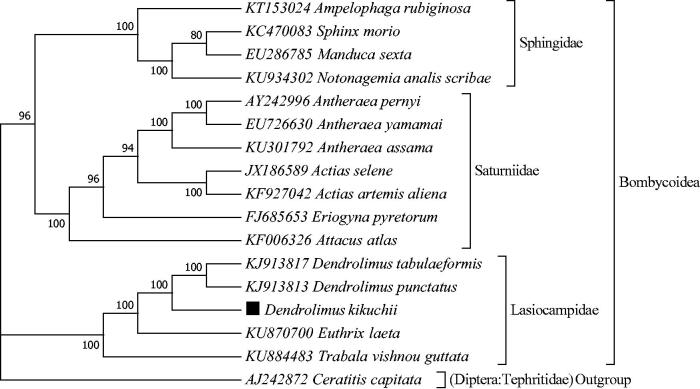
The maximum likelihood (ML) phylogenetic tree of *Dendrolimus kikuchii* and other moths in the superfamily of Bombycoidea. Genbank accessions are indicated with the scientific name of species. The phylogenetic position of *D. kikuchii* was marked in solid square shape. The percentage of replicate trees in which the associated species clustered together in the bootstrap test (500 replicates) is shown next to the branches.
